# Low‐rank inversion reconstruction for through‐plane accelerated radial MR fingerprinting applied to relaxometry at 0.35 T

**DOI:** 10.1002/mrm.29244

**Published:** 2022-04-10

**Authors:** Nikolai J. Mickevicius, Carri K. Glide‐Hurst

**Affiliations:** ^1^ Department of Human Oncology University of Wisconsin‐Madison Madison Wisconsin USA

**Keywords:** CAIPIRINHA, low‐field, MR fingerprinting, non‐Cartesian, parallel imaging, quantitative imaging

## Abstract

**Purpose:**

To reduce scan time, methods to accelerate phase‐encoded/non‐Cartesian MR fingerprinting (MRF) acquisitions for variable density spiral acquisitions have recently been developed. These methods are not applicable to MRF acquisitions, wherein a single k‐space spoke is acquired per frame. Therefore, we propose a low‐rank inversion method to resolve MRF contrast dynamics from through‐plane accelerated Cartesian/radial measurements applied to quantitative relaxation‐time mapping on a 0.35T system.

**Methods:**

An algorithm was implemented to reconstruct through‐plane aliased low‐rank images describing the contrast dynamics occurring because of the transient‐state MRF acquisition. T_1_ and T_2_ times from accelerated acquisitions were compared with those from unaccelerated linear reconstructions in a standardized system phantom and within in vivo brain and prostate experiments on a hybrid 0.35T MRI/linear accelerator.

**Results:**

No significant differences between T_1_ and T_2_ times for the accelerated reconstructions were observed compared to fully sampled acquisitions (*p* = 0.41 and *p* = 0.36, respectively). The mean absolute errors in T_1_ and T_2_ were 5.6% and 2.9%, respectively, between the full and accelerated acquisitions. The SDs in T_1_ and T_2_ decreased with the advanced accelerated reconstruction compared with the unaccelerated reconstruction (*p* = 0.02 and *p* = 0.03, respectively). The quality of the T_1_ and T_2_ maps generated with the proposed approach are comparable to those obtained using the unaccelerated data sets.

**Conclusions:**

Through‐plane accelerated MRF with radial k‐space coverage was demonstrated at a low field strength of 0.35 T. This method enabled 3D T_1_ and T_2_ mapping at 0.35 T with a 3‐min scan.

## 1 INTRODUCTION

Quantitative MRI (qMRI), whose rationale and applications are thoroughly reviewed in Gulani and Seiberlich,^1^ has been demonstrated to be profoundly useful for the objective identification, characterization, and assessment of disease response to treatment. Quantitative tissue properties such as the longitudinal and transverse magnetization relaxation rates (T_1_ and T_2_, respectively) have been used for a variety of applications including assessment of liver cirrhosis severity,^2^ classifying pancreatic lesions,^3^ assessing the severity of renal and prostate cancers,^4^, ^5^ and assessing the response of brain tumors to treatment.^6^, ^7^, ^8^, ^9^ Unfortunately, quantifying T_1_ and T_2_ times with high degrees of precision is challenging due to excessively long scan times. Magnetic resonance fingerprinting (MRF)^10^, ^11^ is a novel approach to qMRI that allows for the rapid and reliable estimation of T_1_ and T_2_. Given its efficiency compared with conventional relaxometry methods,^12^ MRF has been proposed for the serial monitoring of response to radiation therapy treatment on integrated MRI/linear accelerator (MR‐linac) devices.^13^, ^14^

These hybrid imaging and treatment devices are not always equipped with high‐performance hardware seen on diagnostic systems. One such example is the 0.35T MR‐linac (MRIdian; ViewRay, Oakwood Village, OH),^15^ in which the inherently low SNR and weak gradient system (18‐mT/m maximum strength) limits the use of the commonly adopted variable‐density spiral readout for MRF. Therefore, radial k‐space coverage is used to keep the TR—and thus the scan time—short for a fixed MRF pattern length.^14^ Although low‐rank inversion methods have proven to be exceptionally useful for recovering MRF contrast dynamics from a single k‐space spoke per point along the MRF pattern,^13^, ^16^, ^17^ no methods have been proposed to date that recover contrast‐resolved images from through‐plane accelerated radial MRF acquisitions. Having such a capability would enable substantially shorter scan times, thereby minimizing the chance of patient motion corrupting the multiparametric qMRI measurements, while also enabling more efficient integration into the standard radiation therapy workflow where high‐frequency imaging is possible (eg, daily or weekly).

On top of the already highly in‐plane accelerated MRF acquisitions (eg, 48 times below the Nyquist limit^10^), several approaches exist to further accelerate MRF along the through‐plane direction. For simultaneous multislice (SMS) 2D MRF, the flip‐angle pattern of several simultaneously excited slices can be controlled independently, and a pattern‐matching algorithm has been used to separate the aliased slices without the use of parallel imaging methods.^18^ Another SMS 2D‐MRF approach first applies a parallel‐imaging algorithm to separate the heavily undersampled images from each slice before performing a conventional MRF dictionary–matching procedure.^19^ For 3D‐MRF acquisitions using variable‐density spiral readouts, controlled aliasing in parallel imaging (CAIPI or CAIPIRINHA) uniform undersampling along the phase‐encoded direction can be used.^20^ Following an inverse Fourier transform along the undersampled phase‐encoded direction, through‐plane aliasing is present, although its effects are minimal due to the destructive interference of all but one of the aliased slice locations. In this approach, dictionary matching can accurately recover volumetric quantitative parameter maps despite no parallel‐imaging algorithms being used here. For applications in which 3D phase‐encoded radial (ie, “stack of stars”) k‐space coverage is necessary as described previously, this approach will be limited in use due to the extreme in‐plane undersampling levels used when only acquiring a single k‐space spoke per MRF frame. To reconstruct such an MRF acquisition, one potential solution includes a combination of CAIPIRINHA through‐plane sampling and temporal low‐rank constraints to adequately resolve the contrast dynamics in each voxel.

In this work, a low‐rank inversion method to resolve MRF contrast dynamics from through‐plane accelerated acquisitions using the CAIPIRINHA technique will be implemented. The focus will be on uniformly accelerated 3D‐MRF acquisitions, although this algorithm will generalize to SMS 2D approaches. The overall goal of this work is to demonstrate that similar T_1_ and T_2_ times can be obtained from through‐plane accelerated MRF with in‐plane radial k‐space coverage when compared with those that are fully sampled along the through‐plane dimension and reconstructed with a previously validated MRF pipeline on a low‐field (ie, 0.35 T) MRI‐guided radiation therapy system.

## 2 METHODS

### 2.1 Reconstruction formulation

**FIGURE 1:**
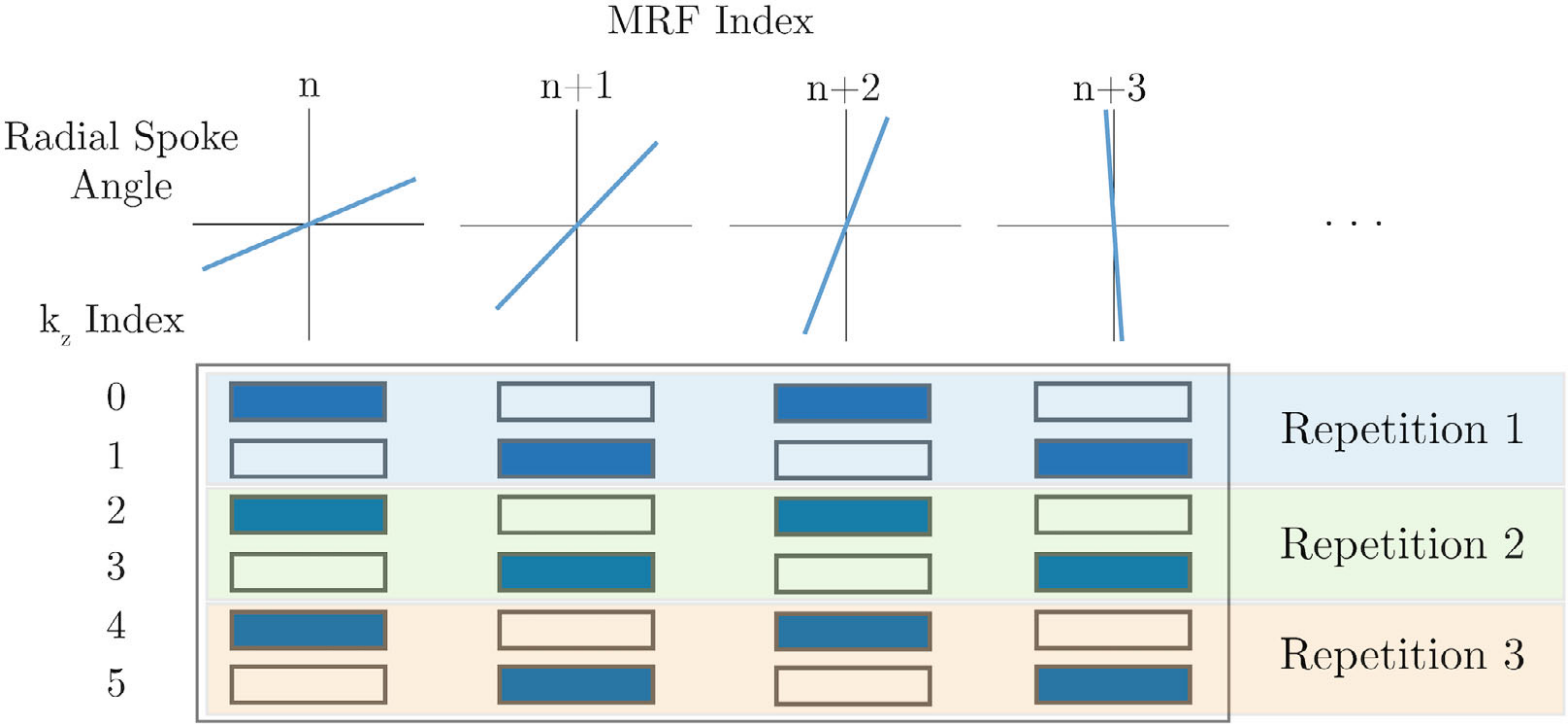
k‐t undersampling scheme for accelerated stack‐of‐stars MR fingerprinting (MRF). At each MRF Index, *n*, along the variable flip‐angle pattern, a unique spoke angle is acquired by incrementing the angle by a tiny golden‐angle increment. For 2‐times through‐plane accelerated acquisition as shown here, the phase‐encoding index (k_z_) alternates between the nearest even and odd sampling index for a given repetition. Within each MRF index, uniform k‐space sampling along k_z_ is achieved. The shift in k_z_ index between repetitions imparts a phase modulation according to the Fourier shift theorem that non‐Cartesian controlled aliasing in parallel imaging (CAIPIRINHA) reconstruction methods exploit to improve image quality

A k‐t undersampling scheme can be seen for an acceleration factor of two (*R* = 2) in Figure 1. After performing k‐t sampling along the phase‐encoding direction of a hybrid Cartesian/radial (ie, stack of stars) MRF acquisition and applying a frame‐by‐frame inverse Fourier transform along the undersampled dimension, the result is a slice‐dependent phase that is modulated throughout the MRF train. For SMS imaging, as demonstrated by Yutzy et al, this phase modulation can be exploited to improve the reconstruction of the aliased slices.^21^ When a uniform through‐plane acceleration factor R is used, the R aliased 2D + contrast images that are to be reconstructed are given by $x\in {\mathbb{C}}^{N\cdot N\cdot R\times T}$, where N is the in‐plane matrix size and T is the number of contrasts. The condition of the inverse problem to recover x is improved when constraining the images to span a low‐dimensional temporal subspace $\Phi \in {\mathbb{C}}^{T\times K}$ with K≪T. Thus, we aim to recover $\alpha \in {\mathbb{C}}^{N\cdot N\cdot R\times K}$ by minimizing the cost function shown in Eq. 1 as follows:

(1) $$\min_{\alpha }{\left\|\mathrm{S\Theta}F\Phi \mathrm{C\alpha}-y\right\|}_{2}^{2}+\lambda \sum_{r}{\left\|R_{r}\left(\alpha \right)\right\|}_{*},$$

where, C represents a multiplication with the coil sensitivities; F is the nonuniform fast Fourier transform operator; Θ is the non‐Cartesian CAIPIRINHA^21^ phase modulation operator that takes into account the frame‐by‐frame phase differences imparted by the k‐t undersampling pattern; and S is an operator that sums over the aliased slice locations. In practice, the gridding weights as part of F are combined with the subspace projection matrix Φ into a single sparse matrix, as described in detail in Assländer et al.^16^ The nuclear norm term (${\left\|\cdot \right\|}_{*}$) imposes locally low rank (LLR) regularization as described in detail by Tamir et al, to improve the conditioning of the inverse problem.^22^ In this term, $R_{r}$ extracts a patch from each subspace coefficient image at position r and rearranges the patch as a column of a matrix.

### 2.2 Data collection

All experiments in this work were performed on a clinical low‐field (0.35 T) MRI‐guided radiation therapy system (MRIdian). An adiabatic inversion‐prepared fast imaging with steady‐state precession MRF sequence was implemented with stack‐of‐stars k‐space coverage and a 512‐point flip‐angle pattern shown in our previous work.^14^ Coronal stack‐of‐stars MRF data were acquired in the National Institute of Standards and Technology/International Society for Magnetic Resonance in Medicine system phantom^23^ using a 12‐channel torso coil array with a matrix size of 128 × 128 × 32 and spatial resolution of 1.64 × 1.64 × 5 mm. To match in vivo data, slice oversampling of 25% was used, bringing the total number of slice phase‐encoding partitions to 40. The TR was 12 ms, and a 3‐s delay was included between subsequent repetitions of the MRF pattern to allow longitudinal magnetization relaxation toward thermal equilibrium. Relaxation occurring during the 3‐s delay between repetitions was taken into account during the calculation of the dictionary. A single radial k‐space spoke was acquired in each MRF frame. Fully partition‐encoded (*R* = 1) MRF was acquired along with a prospectively 2‐times accelerated (ie, *R* = 2) MRF scan with through‐plane reduction factor of *R* = 2. The scan times for each of these acquisitions were 6 min and 3 min, respectively.

Fully partition‐encoded in vivo MRF data sets were acquired in healthy volunteers in the axial orientation in the brain and pelvis of an institutional review board–approved study. The 10‐channel head/neck coil was used for signal reception for the brain scan, and the 12‐channel torso array was used for the pelvis scan. An in‐plane matrix size of 256 × 256 with FOV of 300 × 340 mm were used for the brain and head/neck scans, respectively. The brain and pelvis data were acquired with 40 phase‐encoding partitions and 3‐mm slice thickness. This included 25% oversampling to eliminate aliasing from imperfect slab‐selective excitation pulses. Timing parameters were the same as in the phantom experiments. A single radial k‐space spoke was acquired per frame. The fully sampled data sets were retrospectively undersampled along the phase‐encoding dimension to *R* = 2.

### 2.3 Reconstruction implementation

The inverse problem in Eq. 1 is solved via an alternating direction method of multipliers^24^ algorithm in *Python* on a standalone laptop. An alternating direction method of multipliers solver with LLR regularization has been studied in the context of MRF.^25^ The algorithm was implemented using PyTorch functions to facilitate running the reconstructions on a GPU. The subspace‐constrained nonuniform Fourier transform was implemented using a modified version of the torchkbnufft library^26^ following methodologies by Assländer et al.^16^ The source code to perform these reconstructions along with some sample data has been made publicly available at https://github.com/nmickevicius/mrfCaipiNLM_MRM. The MRF flip‐angle pattern used in these studies has been described in detail previously^14^ and is available for download from this repository. Coil sensitivity maps were estimated with ESPIRiT^27^ using the SigPy library.^28^ The dimensionality of the temporal subspace was experimentally set to *K* = 5. The *R* = 1 data sets were reconstructed using 20 iterations of a linear conjugate gradient algorithm (ie, without regularization), as this was previously demonstrated to agree well with ground‐truth spin‐echo measurements at 0.35 T.^14^ The *R* = 2 reconstructions were performed using eight alternating direction method of multipliers iterations, five conjugate gradient iterations, LLR patch sizes of 8 × 8 pixels, and $\lambda =1\times 10^{-4}$. These parameters were all chosen experimentally. To mitigate additional parallel‐imaging noise arising from the nondiagnostic quality RF coil arrays on the low‐field system, a nonlocal means (NLM) denoising algorithm^29^ was applied slice‐wise to the *R* = 2 subspace coefficient images with a patch size of 3 × 3 pixels and neighborhood search size of 32 × 32 pixels. The NLM was implemented in C with a *MATLAB* (The MathWorks, Natick, MA) mex interface. A dot product‐based dictionary‐matching procedure was performed to map T_1_ and T_2_ using the dictionary from Mickevicius et al^14^ while ignoring $B_{1}^{+}$ variations. although the objective of this work is to ensure that the proposed accelerated MRF processing pipeline yields the same results as the previously validated linear *R* = 1 reconstructions, the *R* = 1 data sets were also reconstructed using LLR regularization and NLM denoising. These data are shown in the Supporting Information. Reconstruction times were recorded for a 2.3‐GHz 4‐Core i7 CPU (Intel, Santa Clara, CA) and a 12GB K80 GPU (NVIDIA, Santa Clara, CA).

### 2.4 Data analysis

For each of the phantom reconstructions, the mean and SD of the T_1_ and T_2_ times estimated in each contrast sphere in the NiCl_2_ contrast plate of the phantom. Paired *t*‐tests were used to test for significant differences in the mean and SDs of the T_1_ and T_2_ times between *R* = 1 and *R* = 2 MRF data sets following Kolmogorov–Smirnov tests for normality. The mean absolute percent error in T_1_ and T_2_ was also calculated. Correlation values between the full and accelerated T_1_ and T_2_ values were calculated. For the in vivo data sets, T_1_ and T_2_ times were extracted within freely drawn regions of interest (ROIs) containing between 14 and 25 voxels from white matter, gray matter, central zone of the prostate, musculoskeletal tissue, and the bone marrow from the in vivo MRF *R* = 1 and *R* = 2 data sets. T_1_ and T_2_ times from the *R* = 1 and *R* = 2 reconstructions from all anatomical ROIs were pooled for further analysis. Correlation coefficients between *R* = 1 and *R* = 2 data sets were calculated, and a paired t‐test was used to test for significant differences between the full and accelerated T_1_ and T_2_ times in vivo following a Kolmogorov–Smirnov test for normality.

## 3 RESULTS

**FIGURE 2:**
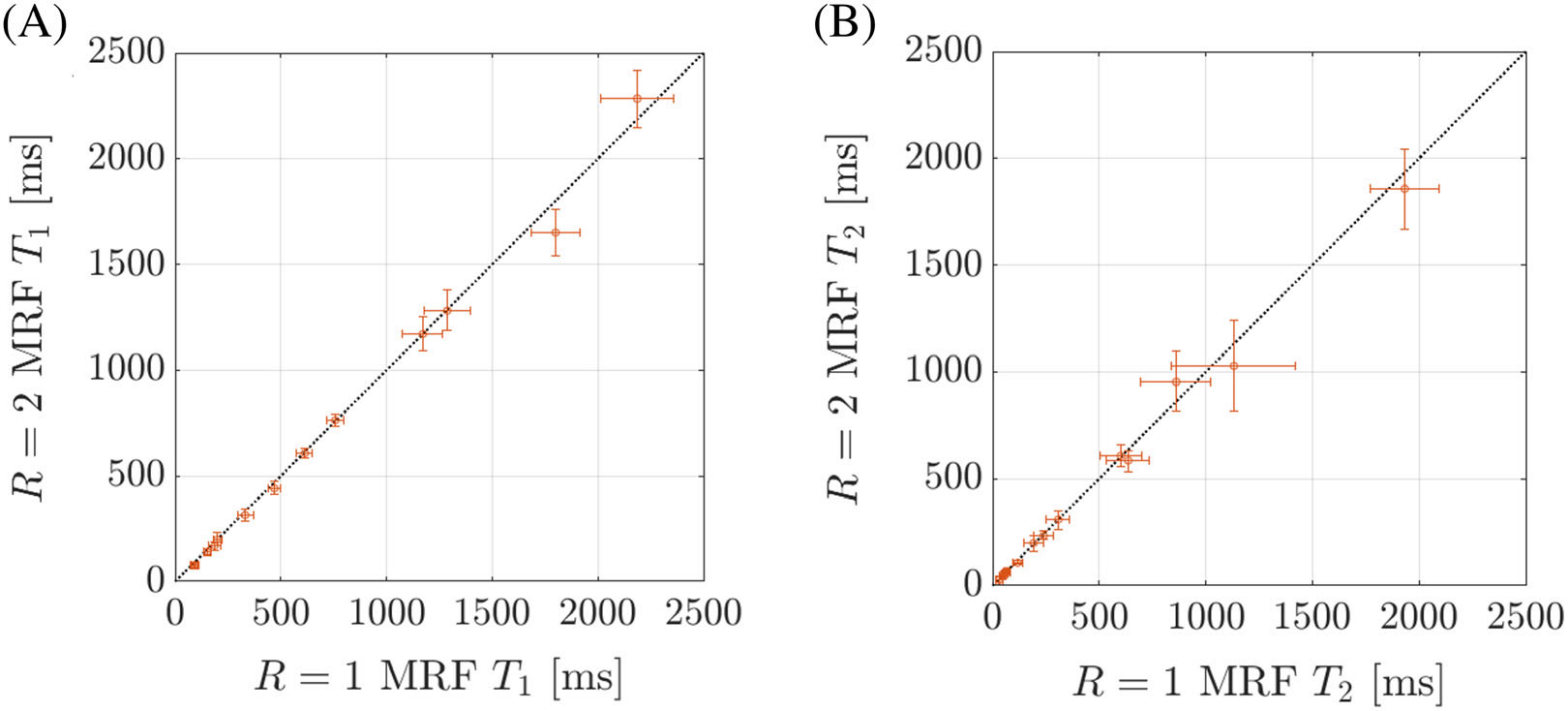
Differences in T_1_ and T_2_ between the fully partition‐encoded (*R* = 1) stack‐of‐stars MRF and the two‐times accelerated (*R* = 2) data sets. The mean values are shown along with SD within each sphere of the National Institute of Standards and Technology/International Society for Magnetic Resonance in Medicine system phantom. The identity line is plotted for reference

Numerical results from the phantom MRF experiments are shown in Figure 2. The means of the T_1_ and T_2_ values from the *R* = 2 reconstructions were found to be very similar to those from *R* = 1, as evidenced by the proximity of the scatter plot points to the identity line. In general, for *R* = 2, low T_1_ times (eg, < 1000 ms) and low T_2_ times (< 500 ms) exhibited better agreements with *R* = 1 values, with lower variability (reduced within‐vial SDs) than higher T_1_ and T_2_ times. Quantitatively, near‐perfect correlations between the fully sampled and accelerated MRF acquisitions were observed for both the T_1_ (0.997) and T_2_ (0.999) times. The use of a parametric *t*‐test was found to be appropriate via the Kolmogorov–Smirnov test, and no significant differences between the *R* = 1 and *R* = 2 acquisitions were observed (*p* = 0.41 for T_1_ and *p* = 0.36 for T_2_). The mean absolute percent error in T_1_ and T_2_ were 5.6% and 2.9%, respectively, between *R* = 1 and *R* = 2 reconstructions. The SDs in T_1_ and T_2_ time estimates for *R* = 2 reconstructions were significantly lower than those from the linear *R* = 1 reconstructions (*p* = 0.02 and *p* = 0.03, respectively). On average, the noise within the *R* = 2 reconstructions for T_1_ and T_2_ were 4.4 times and 3.4 times lower, respectively, than the noise within the *R* = 1 reconstructions. It took 58 s (CPU) or 22 s (GPU) to perform a linear reconstruction for a single slice from the *R* = 1 data sets, and 215 s (CPU) or 50 s (GPU) to perform the alternating direction method of multipliers reconstruction for a pair of slices from the *R* = 2 data sets.

**FIGURE 3:**
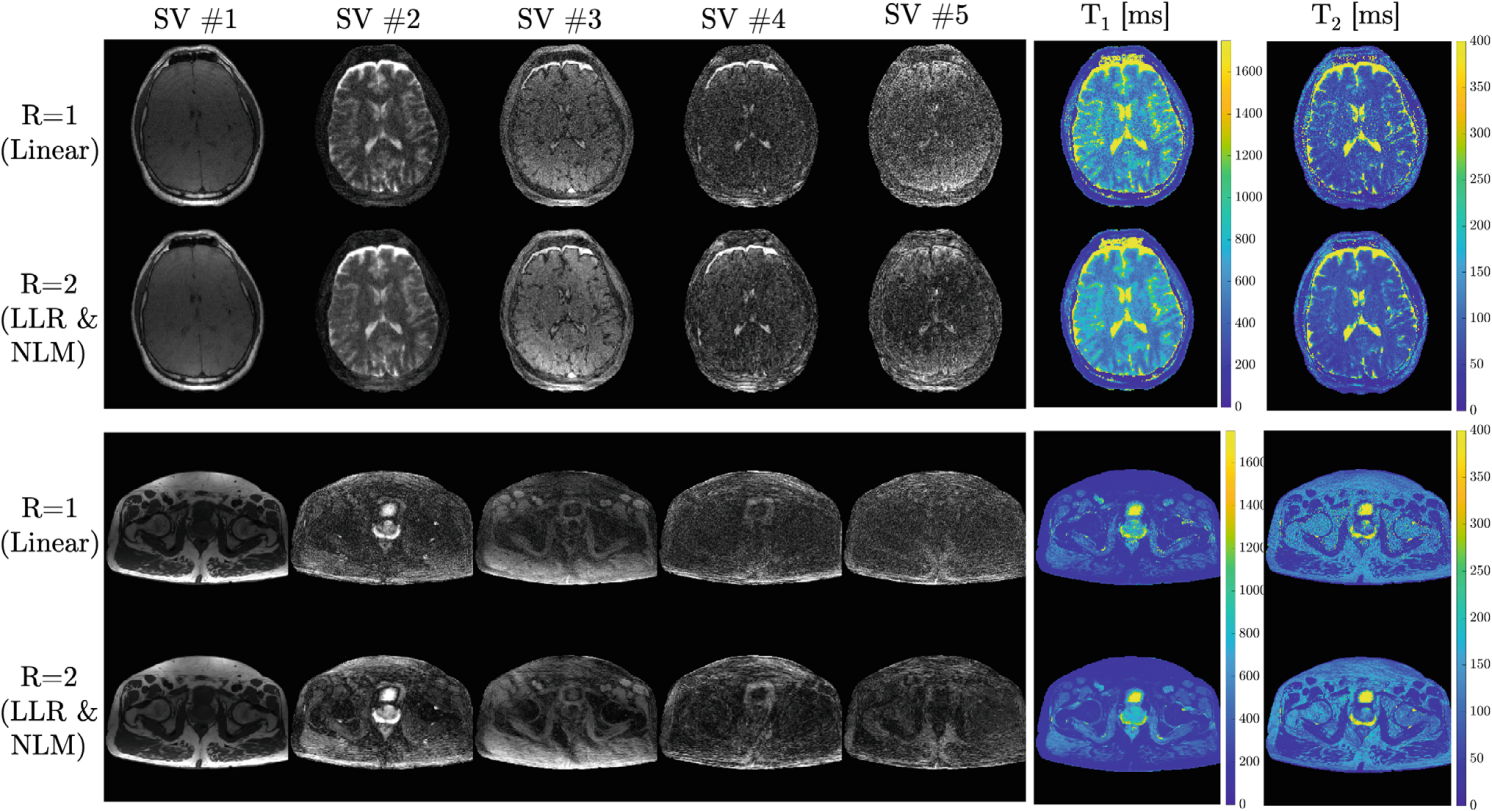
Brain and pelvis in vivo results for fully sampled (*R* = 1) linear reconstruction and accelerated (*R* = 2) locally low rank (LLR) regularized reconstructions with nonlocal means (NLM) denoising. The singular value (SV) images are shown for each data set. Note that each image is windowed individually. T_1_ and T_2_ maps fit via matching of the SV images and the dictionary are shown on the right

**FIGURE 4:**
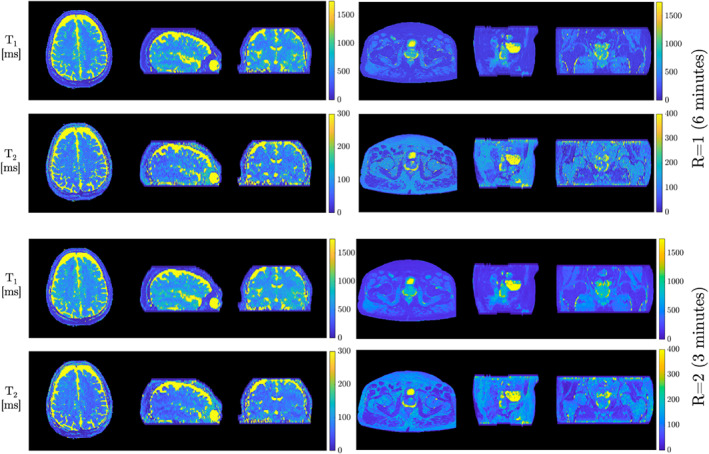
Axial, sagittal, and coronal views of the 3D T_1_ and T_2_ maps from the *R* = 1 and *R* = 2 MRF acquisitions. Comparable image quality of the T_1_ and T_2_ maps can be seen between *R* = 1 and *R* = 2 reconstructions

The singular‐value (SV) images from the subspace constrained reconstruction as well as T_1_ and T_2_ maps for a single slice of the 3D brain and pelvis in vivo experiments are shown in Figure 3. The *R* = 2 SV images, which incorporated LLR regularization and NLM denoising, are visually comparable to the linear *R* = 1 reconstructions. The T_1_ maps were less noisy for the proposed *R* = 2 reconstruction than the linear *R* = 1 reconstruction, with SDs in a white‐matter ROI of 59.6 ms and 36.1 ms for *R* = 1 and *R* = 2, respectively. Likewise, SDs in T_2_ of 25.1 ms and 12.7 ms for *R* = 1 and *R* = 2, respectively, demonstrate that the proposed approach produces T_2_ maps less noisy than the established linear *R* = 1 reconstruction. Figure 4 shows a slice of the natively acquired axial plane and reformatted sagittal and coronal planes of the T_1_ and T_2_ maps for the brain and pelvis data sets. These data demonstrate the capability of the *R* = 2 reconstruction to yield high‐quality T_1_ and T_2_ maps visually comparable to those from the established linear *R* = 1 reconstruction.

**FIGURE 5:**
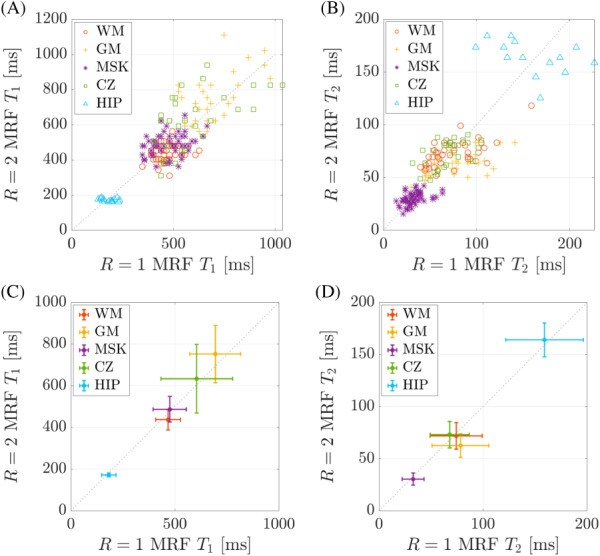
In vivo T_1_ and T_2_ time comparison between fully sampled (*R* = 1) and accelerated (*R* = 2) data sets within regions of interest (ROIs) in white matter (WM), gray matter (GM), musculoskeletal (MSK) tissue, the central zone (CZ) of the prostate, and in the hip. The top row (A,B) shows the T_1_ and T_2_ values from every voxel within all ROIs. The bottom row (C,D) shows the mean T_1_ and T_2_ values along with their SDs within each of these ROIs

The quantitative results from the in vivo MRF data sets are shown in Figure 5. The top row of Figure 5A,B shows the T_1_ and T_2_ values for *R* = 2 against those from *R* = 1. Strong correlations between *R* = 1 and *R* = 2 relaxometry estimates were observed from these data, with correlation coefficients of 0.83 and 0.85 for T_1_ and T_2_, respectively. The mean relaxation times within each ROI were then calculated and used for statistical comparison (Figure 5C,D). No significant differences were observed between *R* = 1 and *R* = 2 measurements with *p* = 0.12 for T_1_ and *p* = 0.58 for T_2_.

## 4 DISCUSSION

This work describes a method to reconstruct through‐plane accelerated MRF quantitative parameter maps. Phantom and in vivo experiments demonstrated that the proposed through‐plane accelerated MRF reconstruction with acceleration factors of *R* = 2 yield similar T_1_ and T_2_ estimates as linear reconstructions from unaccelerated MRF data sets. The overall image quality of the *R* = 2 reconstructions produced with LLR regularization and NLM denoising were less noisy than those produced using a previously validated linear reconstruction for *R* = 2 data sets while maintaining mean T_1_ and T_2_ relaxation times.

The proposed reconstruction pipeline for *R* = 2 included LLR regularization to improve the conditioning of the inverse problem and the additional NLM denoising step. T_1_ and T_2_ times mapped from these reconstructions were compared with a simple linear reconstruction for *R* = 1. This was done because only the linear reconstruction had been previously validated^14^ and demonstrated to be accurate relative to ground‐truth measurements. The image quality of the *R* = 1 reconstructions improves if regularization and NLM denoising was used. A demonstration of this can be seen in Supporting Information Figures S1 and S2.

In the phantom and in vivo experiments, it was observed that tissues with longer T_2_ times exhibit larger SDs. This effect may limit the applicability of MRF—in its present implementation at 0.35 T—for the characterization of long‐T_2_ tissues such as CSF and edema.^10^, ^30^ Optimizing the flip‐angle pattern and timing of the MRF acquisition to minimize the variance in the estimation of T_1_ and T_2_ for a range of expected relaxation times may reduce the uncertainty and improve the reliability of MRF.^17^, ^31^, ^32^ Although this effect is present in the both the accelerated and unaccelerated acquisitions, improving the optimality of the MRF experiment at 0.35 T remains an active area of investigation to reduce uncertainty in the characterization of long‐T_2_ tissues. Every effort to minimize uncertainty in quantitative parameter mapping with MRF will be made to ensure its reliability when using qMRI to characterize, predict, or assess a tumor's response to treatment on low‐field MR‐guided radiation therapy systems.

We were successful in reducing the scan time required for MRF by a factor of 2 in this work, resulting in 3‐min scans to quantify T_1_ and T_2_ relaxation times. Although further reducing scan time would have the benefit of minimizing the likelihood of patient motion during the MRF scans, pushing the rates higher (ie, *R* > =3) results in unusable quantitative maps due excess noise enhancement from the ill‐conditioned reconstruction. While the exact source of the noise enhancement is unknown, future will work will investigate whether this failure to generalize to higher acceleration factors is due to (1) the lower SNR caused by the acquisition of fewer data samples, (2) the modest RF coil encoding (ie, 10–12 channels), or (3) the reliance on exact spoke‐dependent phase differences in the CAIPIRINHA phase operator. Thoroughly assessing the capabilities of this method for acceleration factors greater than 2 at 0.35 T will be a topic of future investigation. The proposed method, however, may be applicable to scanning with higher acceleration rates on higher‐field systems with inherently higher SNR and that are equipped with state‐of‐the‐art RF coil arrays that have a higher channel count.

This initial technical study on a novel algorithm for through‐plane accelerated MRF demonstrated for the acquisition of volumetric T_1_ and T_2_ maps in only 3 min on a 0.35T MR‐linac system. The benefits observed at low magnetic field strength with radial k‐space coverage here may also extend to MRF acquisitions made with more‐common spiral trajectory and higher magnetic field strengths. The technologies proposed here will allow for the acquisition of longitudinal multi‐parametric quantitative imaging throughout the course of radiation therapy treatment, while minimizing the burden of excessive scan times.

## Supporting information


**Figure S1**. Brain and pelvis in vivo results for fully sampled (*R* = 1) reconstruction and accelerated (*R* = 2) reconstruction. Both reconstructions use locally low rank (LLR) regularization with non‐local means (NLM) denoising. The singular value (SV) images are shown for each dataset. Note that each image is windowed individually. T_1_ and T_2_ maps fit via matching of the SV images and the dictionary are shown on the right
**Figure S2**. Axial, sagittal, and coronal views of the 3D T_1_ and T_2_ maps from the *R* = 1 and *R* = 2 MR fingerprinting (MRF) acquisitions. Comparable image quality of the T_1_ and T_2_ maps can be seen between *R* = 1 and *R* = 2 reconstructions. Both reconstructions used LLR regularization and NLM denoising
