# Supplementary material for: Low‐rank inversion reconstruction for through‐plane accelerated radial MR fingerprinting applied to relaxometry at 0.35 T
Source: Magn Reson Med. 2022 Apr 10;88(2):840–8. doi: 10.1002/mrm.29244 (PMC9324087; doi:10.1002/mrm.29244)
Supplement: Supplementary file 1 — Figure S1. Brain and pelvis in vivo results for fully sampled (R = 1) reconstruction and accelerated (R = 2) reconstruction. Both reconstructions use locally low rank (LLR) regularization with non‐local means (NLM) denoising. The singular value (SV) images are shown for each dataset. Note that each image is windowed individually. T1 and T2 maps fit via matching of the SV images and the dictionary are shown on the right Figure S2. Axial, sagittal, and coronal views of the 3D T1 and T2 maps from the R = 1 and R = 2 MR fingerprinting (MRF) acquisitions. Comparable image quality of the T1 and T2 maps can be seen between R = 1 and R = 2 reconstructions. Both reconstructions used LLR regularization and NLM denoising [file MRM-88-840-s001.docx]

**
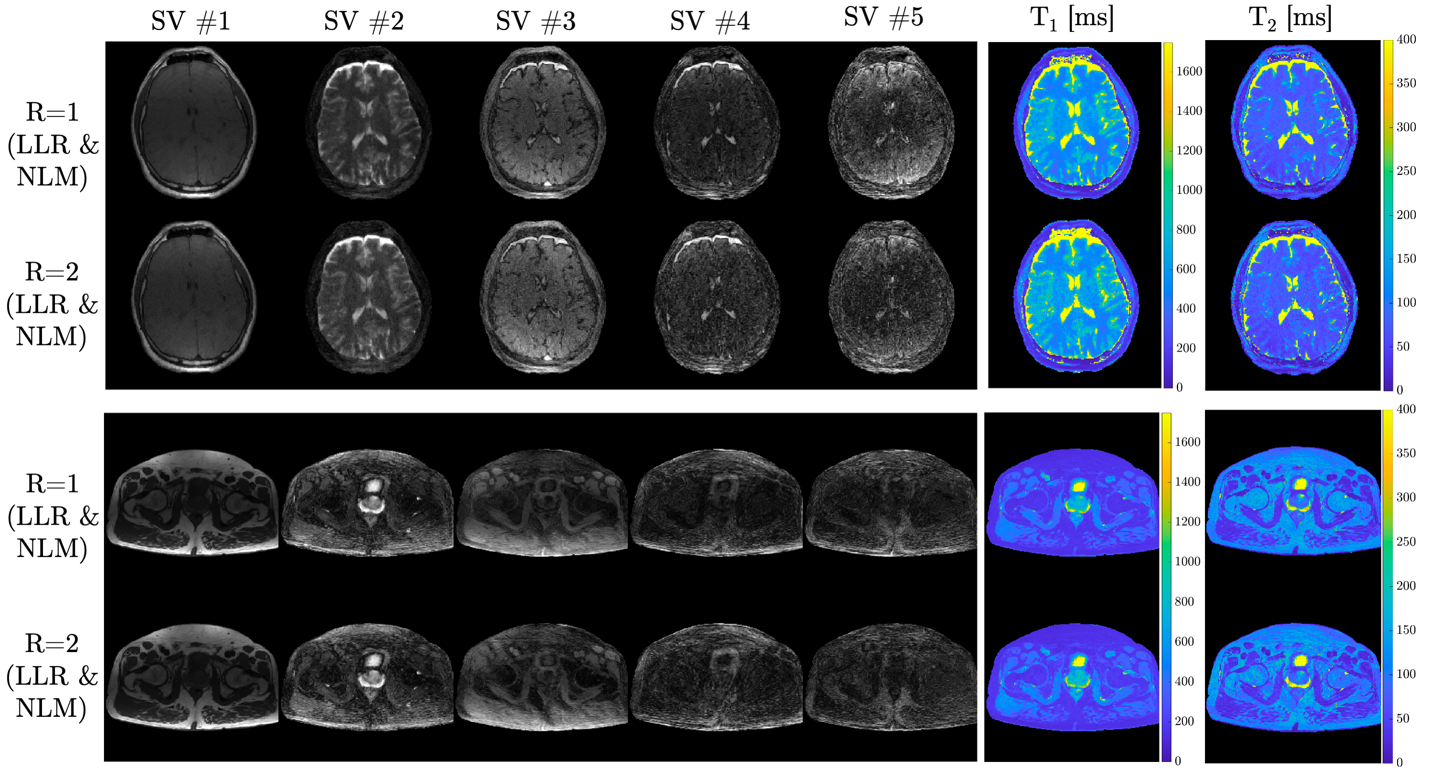
**

**Supporting Information Figure S1**. Brain and pelvis in vivo results for fully sampled (R=1) reconstruction and accelerated (R=2) reconstruction. Both reconstructions employ locally low rank (LLR) regularization with non-local means (NLM) denoising. The singular value (SV) images are shown for each dataset. Note that each image is windowed individually. T_1_ and T_2_ maps fit via matching of the SV images and the dictionary are shown on the right.


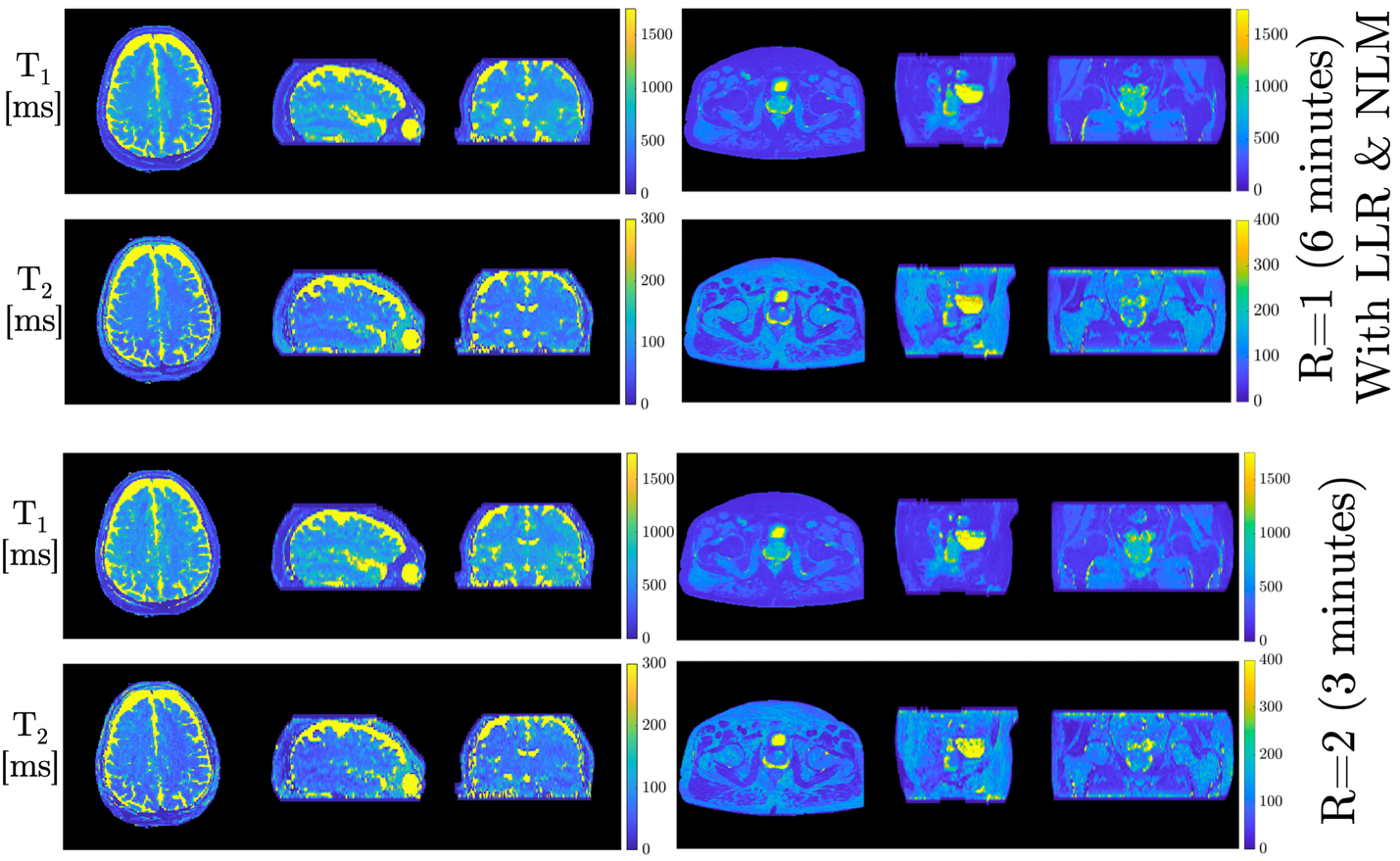


**Supporting Information Figure S2**. Axial, sagittal, and coronal views of the 3D T_1_ and T_2_ maps from the R=1 and R=2 MRF acquisitions. Comparable image quality of the T_1_ and T_2_ maps can be seen between R=1 and R=2 reconstructions. Both reconstructions employed locally low rank (LLR) regularization and non-local means (NLM) denoising.
